# The aminoshikimic acid pathway in bacteria as source of precursors for the synthesis of antibacterial and antiviral compounds

**DOI:** 10.1093/jimb/kuab053

**Published:** 2021-08-10

**Authors:** Adelfo Escalante, Rubén Mendoza-Flores, Guillermo Gosset, Francisco Bolívar

**Affiliations:** Departamento de Ingeniería Celular y Biocatálisis, Instituto de Biotecnología. Universidad Nacional Autónoma de México, Av. Universidad 2001, Colonia Chamilpa, 62210, Cuernavaca, Morelos, México; Departamento de Ingeniería Celular y Biocatálisis, Instituto de Biotecnología. Universidad Nacional Autónoma de México, Av. Universidad 2001, Colonia Chamilpa, 62210, Cuernavaca, Morelos, México; Departamento de Ingeniería Celular y Biocatálisis, Instituto de Biotecnología. Universidad Nacional Autónoma de México, Av. Universidad 2001, Colonia Chamilpa, 62210, Cuernavaca, Morelos, México; Departamento de Ingeniería Celular y Biocatálisis, Instituto de Biotecnología. Universidad Nacional Autónoma de México, Av. Universidad 2001, Colonia Chamilpa, 62210, Cuernavaca, Morelos, México

**Keywords:** Aromatic metabolism, Aminoshikimic acid pathway, AHBA, Metabolic engineering, Antimicrobial, Antiviral compounds

## Abstract

The aminoshikimic acid (ASA) pathway comprises a series of reactions resulting in the synthesis of 3-amino-5-hydroxybenzoic acid (AHBA), present in bacteria such as *Amycolatopsis mediterranei* and *Streptomyces*. AHBA is the precursor for synthesizing the mC_7_N units, the characteristic structural component of ansamycins and mitomycins antibiotics, compounds with important antimicrobial and anticancer activities. Furthermore, aminoshikimic acid, another relevant intermediate of the ASA pathway, is an attractive candidate for a precursor for oseltamivir phosphate synthesis, the most potent anti-influenza neuraminidase inhibitor treatment of both seasonal and pandemic influenza. This review discusses the relevance of the key intermediate AHBA as a scaffold molecule to synthesize diverse ansamycins and mitomycins. We describe the structure and control of the expression of the model biosynthetic cluster *rif* in *A. mediterranei* to synthesize ansamycins and review several current pharmaceutical applications of these molecules. Additionally, we discuss some relevant strategies developed for overproducing these chemicals, focusing on the relevance of the ASA pathway intermediates kanosamine, AHAB, and ASA.

## Introduction

**Fig. 1:**
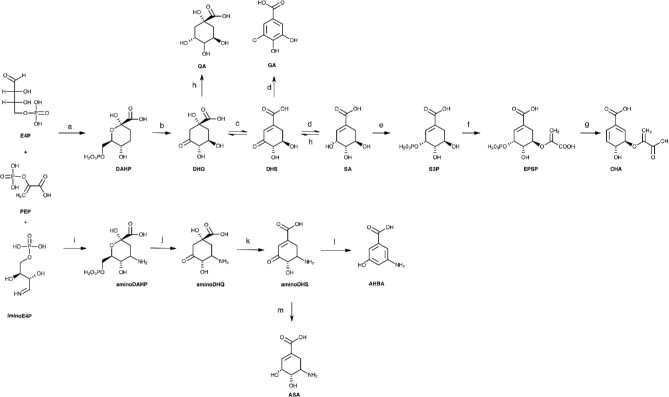
The shikimic and aminoshikimic acids pathway. SA pathway in *Escherichia coli* (upper section). DAHP, 3-deoxy-D-*arabino*-heptulosonate-7-phosphate; DHQ, 3-dehydroquinate; DHS, 3-dehydroshikimate; SA, shikimic acid; S3, SHK-3-phosphate; EPSP, 5-enolpyruvyl-shikimate 3-phosphate; CHA, chorismate. Enzymes and coding genes are indicated. (a) DAHP synthase (AroF, AroG, AroH isoenzymes, *aroF, aroG, aroH, respectively*); (b) DHQ synthase (*aroB*); (c) DHQ dehydratase (*aroD*); (d) SA dehydrogenase (*aroE*); (e) SA kinase I, II isoenzymes (*aroK, aroL*, respectively); (f) EPSP synthase (*aroA*); (g) CHA synthase (*aroC*); (h) SA dehydrogenase/quinate dehydrogenase (*ydiB*). The aminoshikimic acid (ASA) pathway from aminoDAHP to 3-amino-5-hydroxybenzoic acid (AHBA) and ASA in *Amycolatopsis mediterranei* (lower section). (i) aminoDHPS (*rifH*); (j) aminoDHQ synthase (*rifG*); (k) aminoDHS dehydratase (*rifJ*); (l) AHBA synthase (*rifK*); (m) aminoSHK dehydrogenase (*rifI*). E4P, erythrose-4-phosphate; PEP, phosphoenolpyruvate. Adapted from Diaz Quiroz et al. (2014), Guo & Frost (2004), Kang et al. (2012), Martínez et al. (2015).

The biosynthesis of aromatic compounds is an essential metabolic trait present mainly in bacteria, yeasts, and plants. These biosynthetic pathways are involved in synthesizing a high diversity of primary and secondary metabolites with critical cellular functions in prokaryotic and eukaryotic organisms. From the biotechnological point of view, these aromatic metabolites are highly valuable compounds involved in synthesizing structural blocks of proteins and compounds with relevant antiviral, antimicrobial, and anticancer activities, representing a one billion dollar market (Braga & Faria, 2020; Cao et al., 2020; Kallscheuer et al., 2019; Lee et al., 2019; Li et al., 2020; Liu et al., 2020a; Martínez et al., 2019; Shen et al., 2020). Aromatic metabolism occurs mainly through the shikimic acid (SA) pathway present in bacteria, fungi, higher plants, and certain other organisms, but it is absent in mammals (Diaz Quiroz et al., 2014; Li et al., 2020; Martínez et al., 2015). This pathway recruits the carbon flux from the central carbon metabolism pathways through phosphoenolpyruvate (PEP) and erythrose 4-phosphate (E4P), key intermediates from the glycolytic pathway and the pentose phosphate pathway (PPP), respectively, to the synthesis of 3‐deoxy‐D‐arabinoheptulosonate‐7‐phosphate (DAHP), the initial substrate of the SA pathway (Fig. 1). The final product of the SA pathway is the chorismate (CHA), which is, in turn, the common intermediate for the biosynthesis of the three aromatic amino acids (L-phenylalanine, L-tyrosine, and L-tryptophan). Other critical intermediates of the SA pathway, such as dehydroshikimic acid (DHS), SA, and CHA, are key precursors for synthesizing many high-value natural products, including phenylpropanoids and flavonoids produced by plants (Cao et al., 2020; Estevez & Estevez, 2012; Kallscheuer et al., 2019; Li et al., 2020; Shen et al., 2020; Tohge & Fernie, 2017). The aminoshikimic acid (ASA) pathway is present in microorganisms such as *Amycolatopsis mediterranei* and *Actinosynnema pretiosum* as *Streptomyces* species (Floss et al., 2011; Kang et al., 2012). This pathway was considered initially as a variant of the SA pathway because the metabolic intermediates SA, quinic acid (QA), and 3-dehydroquinic acid (DHQ) were proposed as the source of 3-amino-5-hydroxybenzoic acid (AHBA), the precursor for the synthesis of the *meta*-C-C_6_-N (mC_7_N) units, the characteristic structural component of ansamycins and mitomycins antibiotics (Floss, 1997; Hornemann et al., 1974, 1980).

Ansamycin antibiotics are an abundant class of AHBA-derived natural products with valuable pharmaceutical relevance, exhibiting broad biological activities such as antibiotics, anticancer agents, and enzyme inhibitors. Mitomycins are another important class of natural products derived from AHBA and have a relevant anticancer activity. Aminoshikimic acid (5-amino-5-deoxyshikimic acid) is a highly valuable compound with great potential biotechnological applications as with SA, is a precursor in the chemical synthesis of viral neuraminidase inhibitors such as oseltamivir phosphate (OSP) and other potential antiviral inhibitors as 4,5-diamino shikimic acid derivatives (Diaz Quiroz et al., 2014; Floss et al., 2011; Guo & Frost, 2004; Kang et al., 2012; Karpf & Trussardi, 2009). In this review, we discuss the relevance of the ASA pathway as a source of scaffold molecules for the synthesis of various relevant pharmaceutical chemicals. Additionally, we describe the structure of the biosynthetic cluster *rif* in *A. mediterranei* and the mechanisms involved in controlling its expression as an example of the structure and function of a biosynthetic cluster for the synthesis of ansamycins and mitomycins. Finally, we highlight their current and potential pharmaceutical uses and reviews some examples of the application of metabolic engineering strategies in diverse bacterial strains to overproduce the critical intermediates kanosamine, AHBA, and ASA.

## Elucidation of the Aminoshikimic Acid Pathway

Feeding experiments of the SA pathway intermediates SA, QA, and DHQ to synthesize mC_7_N units of ansamycins and mitomycins repeatedly failed in experiments with *A. mediterranei*. Additionally, labeled [2–^13^C]-SA was not incorporated into the C_7_N units to synthesize ansatrienin but efficiently labeled the cyclohexane carboxylic acid moiety, leading to conclude that the biosynthesis of the C_7_N units must branch off from the SA pathway before SA. Further genetic experiments showed that the branching point for the synthesis of C_7_N in the biosynthesis of rifamycin was before DHQ (Haber et al., 2002; Hornemann et al., 1974, 1980; Karlsson et al., 1974; White & Martinelli, 1974).

Based on degradation experiments of mitomycins and geldanamycin labeled with various precursors, it was determined that C-5-equivalents carry the nitrogen of AHBA. This result led to the proposal of the existence of 3,4-dideoxy-4-amino-D-arabino-heptulosonic acid 7-phosphate (aminoDAHP) as the specific precursor of AHBA (Floss et al., 2011; Hornemann et al., 1974, 1980). Identification of glutamine as the nitrogen source for rifamycin biosynthesis suggested that aminoDAHP was synthesized in a reaction independent of E4P, with ammonia from the hydrolysis of glutamine to result in an imine (iminoE4P). IminoE4P is condensed with PEP in a parallel reaction to the synthesis of DAHP in the SA pathway (Diaz Quiroz et al., 2014; Floss, 1997; Floss et al., 2011; Kang et al., 2012). The aminoDAHP fuels the ASA pathway consisting of four parallel reactions to the SA pathway: the synthesis of aminoDAHP, its conversion to aminoDHQ, which is dehydrated to aminoDHS, and then aromatized to AHBA by an AHBA synthase, in a reaction that has no analogous step in the SA pathway (Fig. 1) (Diaz Quiroz et al., 2014; Floss et al., 2011; Kang et al., 2012). Purification of the AHBA synthase enzyme, and cloning of the encoding gene *rifK*, resulted in the further analysis of the entire 95 kbp rifamycin (rif) biosynthetic gene cluster from *A. mediterranei.* The cluster contains the genes *rifGHIKLMN* and *rifJ* involved in the biosynthesis of AHBA: aminoDAHP synthase (RifH), aminoDHQ synthase (RifG), aminoDHQ dehydratase (RifJ), AHBA synthase (RifK); and those genes involved in the synthesis of the precursor iminoE4P for the synthesis of aminoDAHP: an oxidoreductase (RifL), a phosphatase (RifM), and a kinase (RifN) (Arakawa et al., 2002; Diaz Quiroz et al., 2014; Floss et al., 2011; Guo & Frost, 2004; Kang et al., 2012).

### The ASA Pathway in Amycolatopsis mediterranei

**Fig. 2:**
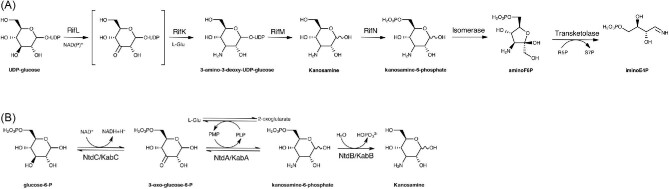
Kanosamine biosynthesis pathway in *Amycolatopsis mediterranei* and several *Bacillus* species. (A) Kanosamine biosynthesis from UDP-glucose in *A. mediterranei* as part of the *rif* cluster. RifL, UDP-3-keto-D-glucose dehydrogenase (*rifL*); RifK, UDP-3-keto-D-glucose transaminase (*rifK*); RifM, UDP-kanosamine phosphatase (*rifM*); RifN, kanosamine kinase (*rifN*) (Arakawa et al., 2002; Guo & Frost, 2004; Kang et al., 2012). (B) Kanosamine biosynthesis from glucose-6-P in *Bacillus pumillus* ATCC21143 (Guo & Frost, 2004), *Bacillus subtilis* (Vetter et al., 2013), and *Bacillus cereus* UW85 (Prasertanan & Palmer, 2019). *kabCAB* operon in *B. cereus* and the homologous *ntdCAB* operon in *B. subtilis.* KabC/NtdC, glucose-6-phosphate 3-dehydrogenase; KabA/NtdA, pyridoxal phosphate-dependent 3-oxo-glucose-6-phosphate:glutamate aminotransferase; KabB/NtdB, kanosamine-6-phosphate phosphatase.

The ASA pathway for the synthesis of AHBA and ASA in *A. mediterranei* consists of two stages, the synthesis of imino-D-erythrose 4-phosphate (iminoE4P) (Fig. 2A) and the synthesis of AHBA and ASA from aminoDAHP. The enzymes involved in the biosynthesis of these metabolites are encoded as part of the *rif* cluster comprising *rifGHIKLMN* and the *rifJ* gene (Floss et al., 2011; Guo & Frost, 2004; Kang et al., 2012). The synthesis of iminoE4P starts from UDP-glucose as the substrate of RifL, an oxidoreductase dependent of NAD(P)^+^ and a transamination catalyzed by RifK (AHBA synthase) with glutamate as the amino donor, resulting in the synthesis of 3-amino-3-deoxy-UDP-glucose. The other activity of RifM (UDP-kanosamine phosphatase) yields kanosamine. RifN (kanosamine kinase) phosphorylates kanosamine resulting in kanosamine-6-phosphate (K6P), which is isomerized by the phosphokanosamine isomerase to amino-D-fructose 6-phosphate (aminoF6P). The aminoF6P and ribose-5-phosphate are converted to iminoE4P and sedoheptulose-7-phosphate (S7P) by the enzyme transketolase (*orf15*) (Arakawa et al., 2002; Floss et al., 2011; Guo & Frost, 2002a,b; Kang et al., 2012) (Fig. 2A).

The iminoE4P and PEP are condensed to aminoDAHP by RifH. This step is followed by three parallel reactions to the SA pathway to yield aminoDHQ, aminoDHS, and AHBA catalyzed by RifG, RifJ, and RifK, respectively. Remarkably, AHBA synthase (RifK) possess two catalytic functions: As a homodimer, it catalyzes the last reaction in the pathway to produce AHBA from aminoDHS, and in the iminoE4P stage, in a complex with the oxidoreductase RifL, catalyzes the transamination of UDP-3-keto-D-glucose to 3-amino-3-deoxy-UDP-glucose (Fig. 2A) (Floss et al., 2011; Kang et al., 2012). Finally, ASA is synthesized by reducing aminoDHS by the activity of the aminoshikimate dehydrogenase encoded by the *rifI* gene in *A. mediterranei* (Fig. 1) (Guo & Frost, 2004).

**Fig. 3:**
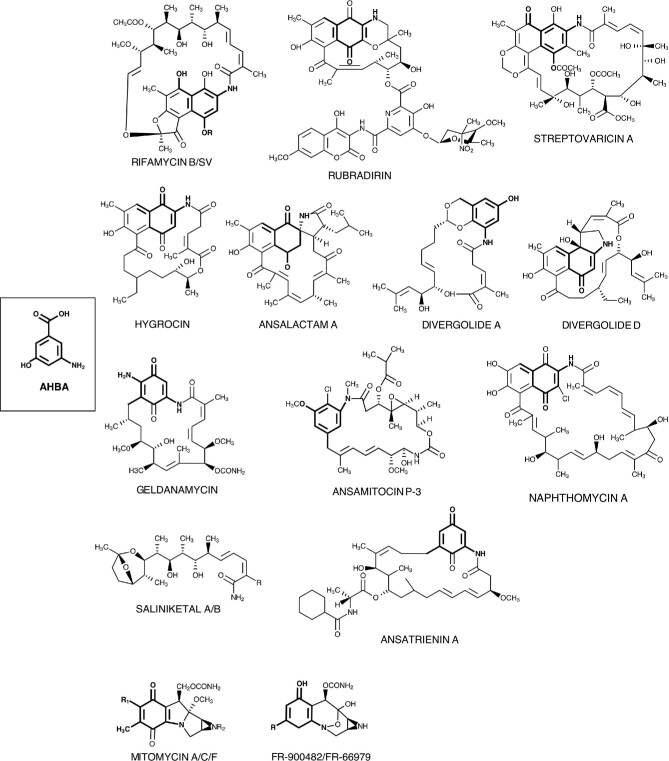
Diversity of derived natural products from 3-amino-5-hydroxybenzoic acid (AHBA). Chemical structure of AHBA shown as reference. Chemical structures of ansamycin derivatives. Chemical structure of mitomycin derivatives. The AHBA moiety is highlighted in bold bonds and atoms in the chemical structures shown. For rifamycin: R = CH_2_COOH for rifamycin B, R = H for rifamycin SV. For saliniketal: R = H for saliniketal A, R = OH for saliniketal B. For mitomycin: R_1_ = OCH_3_ and R_2_ = H for mitomycin A, R_1_ = NH_2_ and R_2_ = H for mitomycin C, R_1_ = OCH_3_ and R_2_ = CH_3_ for mitomycin F. Modified from Kang et al. (2012).

All ansamycin antibiotics are composed of a benzoic or naphthalenic chromophore bridged by an aliphatic polyketide chain that terminates at the chromophore with an amide linkage (Watanabe et al., 2003). The aromatic moiety is derived from an AHBA starter unit activated by a nonribosomal peptide synthetase-like mechanism. The chain extension by subsequent additions of methylmalonyl and malonyl extender units is performed by the activity of modular polyketide synthases (PKSs) (Floss et al., 2011; Kang et al., 2012; Watanabe et al., 2003). A comprehensive review of the biosynthesis of naphtalenic ansamycins—including the rifamycins, streptovaricins, rubradirins, naphthomycins, hygrocins, ansalactam A, chaxamycins, and divergolides is reported by Kang et al. (2012). Mitomycins and the compound FR-900482 are compounds with similar structures produced by diverse *Streptomyces* species and represent a family of antitumor agents of extraordinary potency (Kang et al., 2012). Based on the nature and stereochemistry of the radical at C-9, mitomycins are classified in mitomycin A, C, and F, whereas there are only two members of type FR, FR-900482, and FR-66979. The aromatic rings of the mitomycins are quinones, whereas in FR-900482 are phenols (Judd & Williams, 2004; Kang et al., 2012). FR-900482 was isolated from cultures of *S. sandansis* no. 6897 and possess an azidine and a carbamoylated hydroxymethyl moiety resembling the mitomycins' structure and suggests a common biosynthetic pathway (Kang et al., 2012). FR-900482 possesses an attractive antitumor activity, superior to mitomycin C (Hirai et al., 1987; Shimomura et al., 1987). The structure of FR-900482—showing a unique hydroxylamine hemiacetal—challenged its synthetic synthesis, resulting in its total chemical synthesis and the synthesis of several enantioselective synthesis analogs (Kambe et al., 2001). Fig. 3 shows representative chemical structures of ansamycins and mitomycins derived from AHBA.

### The Biosynthetic Clusters for Rifamycin and Mitomycin Biosynthesis Are Examples of the Genetic Organization of the Biosynthetic Pathways of Ansamycins and Mitomycins

**Fig. 4:**
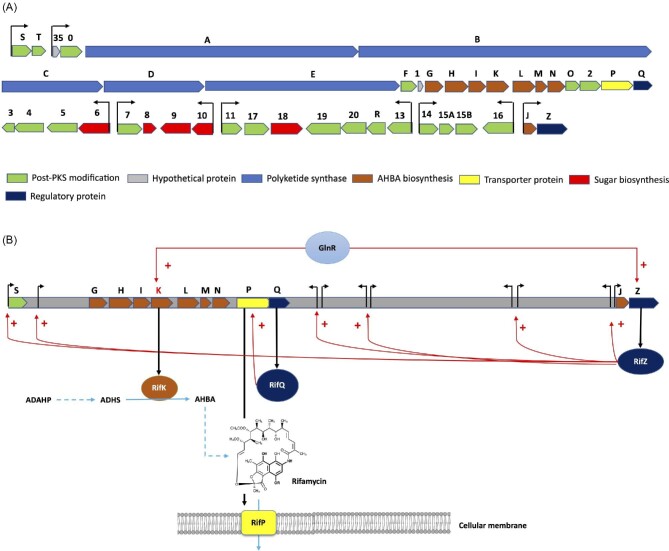
The organization of the *rif* cluster and proposed regulation mechanisms. (A) Schematic representation of the ∼90 kb *rif* cluster of *Amycolatopsis mediterranei* U32 organized in 10 operons encoding for the synthesis of the precursor AHBA; assembly and modification of the polyketide backbone, downstream, conversion, and export of rifamycin; and regulatory expression genes. (B) Schematic representation of the regulation of the *rif* cluster by the global regulator RifZ and proposed indirect regulation of the *rif* cluster by GlnR. The role of GlnR in the direct regulation of the expression of the AHBA synthase enzyme encoded by *rifK*, results in the increased biosynthesis of AHBA. The proposed role of RifQ on the expression of rifamycin transporter (*rifP*). Genes in the *rif* cluster: S, *rifS*; T, *rifT*; 35, orf35; 0, orf0; A, *rifA*; B, *rifB*; C, *rifC*; D, *rifD*; E, *rifE*; F, *rifF*; 1, orf1; G, *rifG*; H, *rifH*; I, *rifI*; K, *rifK*; L, *rifL*; M, *rifM*; N, *rifN*; O, *rifO*; 2, orf2; P, *rifP*; Q, *rifQ*; 3, orf3; 4, orf4; 5, orf5; 6, orf6; 7, orf7; 8, orf8; 9, orf9; 10, orf10; 11, orf11; 17, orf17; 18, orf18; 19, orf19; 20, orf20; R, *rifR*; 13, orf13; 14, orf14; 15A, orf15A; 15B, orf15B; 16, orf16; J, *rifJ*; Z, *rifZ*. ADAHP, aminoDAHP; ADHS, aminoDHS; AHBA, 3-amino-5-hydroxybenzoic acid. ;, : show the start of the 10 operons in the cluster. Red color arrows indicate positive transcription regulation mechanisms. Black color arrows indicate transcription and translation of the corresponding encoding protein. Blue color dashed and continuous lines indicate several and one enzymatic reaction, respectively, in the ASA pathway. Modified from Floss et al. (2011) Liu et al. (2020).

The ∼90-kb rifamycin biosynthetic gene cluster (*rif* cluster) from several *A. mediterranei* and several *Streptomyces* species strains includes 43 genes organized in 10 operons and starts with *rifS* and ends with *rifZ* (Fig. 4A). The cluster includes an operon comprising five types of modular polyketide synthases (*rifABCDE*), associated with the genes involved in the synthesis of AHBA (*rifGHIKLMN*) and *rifJ* (in a separated operon). Additionally, it comprises genes controlling the post-polyketide backbone modifications and conversion of rifamycin and its export (Floss et al., 2011; Li et al., 2017; Liu et al., 2020c). Furthermore, two regulator genes are located in the *rif* cluster, RifZ (*rifZ*), a LuxR family transcriptional regulator activating the transcription of all the genes of the cluster; and RifQ (*rifQ*), proposed as involved in the control of the reduction of the intracellular toxicity of rifamycin by regulating the expression of the rifamycin exporter RifP (*rifP*) (Fig. 4A) (Li et al., 2017; Liu et al., 2020c). Remarkably, besides RifZ, GlnR, a global transcriptional regulator in actinomycetes involved in the modulation of assimilation and utilization of diverse carbon sources; nitrogen sources (ammonium, urea, or nitrate), as in antibiotic biosynthesis, is also involved in the activation of the positive regulator of RifZ, promoting the indirect positive regulation of the entire *rif* cluster. Additionally, GlnR was proposed to promote the biosynthesis of AHBA directly by activating the transcription of *rifK* coding for AHBA synthase (Liu et al., 2020c) (Fig. 4B).

As ansamycins, mitomycins are synthesized from AHBA by the encoded genes in  the ∼55-kb mitomycin biosynthetic cluster, comprising 47 genes for the biosynthesis of mitomycin C (MCC) in *Streptomyces lavendulae*, including homologous to the genes involved in the synthesis of iminoE4P and AHBA in *A. mediterranei* and *Streptomyces coelicolor* in the *rif* cluster, and the genes involved in the synthesis of mitomycin from AHBA and N-acetylglucosamine (GlcNAc) (Bass et al., 2013; Nguyen & Yokoyama, 2019). Furthermore, the coupling mechanism of the building blocks (AHBA and GlcNAc) for mitomycin biosynthesis was recently elucidated. In the proposal mechanism, AHBA is first loaded onto an MmcB acyl carrier protein (ACP) by the activity of MitE (acyl ACP synthetase) followed by a transfer of GlcNAc from UDP-GlcNAc by MitB, suggesting that metabolic intermediates in the early stages in the biosynthesis of mitomycins are coupled to MmcB (Nguyen & Yokoyama, 2019).

### Relevant Biological Activities of Compounds Derived from AHBA

Ansamycins produced by *A. mediterranei* include the rifamycins, from which derive a large number of chemically synthesized compounds, including rifampicin, rifabutin, rifapentine, and rifaximin (Kang et al., 2012; Liu et al., 2020c). These compounds are still used as first-line antimycobacterial drugs against *Mycobacterium tuberculosis* and *Mycobacterium leprae* by inhibiting their RNA polymerase activity (Liu et al., 2020c).

Saliniketal A and B are interesting bicyclic polyketide compounds because of their structural relationship to the *ansa* side chain (C-1 through C-15) of the rifamycin antibiotics (Fig. 3). These compounds were isolated from supernatant cultures of several strains of the marine actinomycetes *Salinispora arenicola*. These compounds possess anticancer activity associated with the possible inhibition of the induction of ornithine decarboxylase (ODC). Inhibition of ODC activity is proposed to decrease polyamines' cellular concentration, considered as an effective strategy to prevent carcinogenesis (Kang et al., 2012; Williams et al., 2007).

The ansamycin antibiotic rubradirin (Fig. 3) isolated from *Streptomyces achromogenes* var. *rubradiris* NRRL3061 shows antimicrobial activity against many Gram-positive bacteria, including multiple antibiotic-resistant strains *Staphylococcus aureus.* The structures of rubradirins consist of four covalently linked distinct moieties, including rubransarol, 3-amino-4-hydroxy-7-methoxycoumarin, 3,4-dihydrodipicolinate, and D-rubranitrose (2,3,6-trideoxy-3-*C*-4-*O*-dimethyl-3-*C*-nitro-D-*xylo*-hexose). These moieties are proposed as critical for the antibacterial activity of these ansamycin antibiotics (Kang et al., 2012). Rubradirin inhibits translation chain initiation during protein synthesis at the bacterial ribosome, and the derivative rubradirin aglycone shows inhibition against bacterial RNA polymerase by a different mechanism from those reported for other ansamycins. Additionally, rubradirin aglycone acts as a potent inhibitor of the human immunodeficiency virus (HIV) reverse transcriptase (Boll et al., 2011; Kim et al., 2008).

Naphthomycins have the longest aliphatic *ansa* carbon chain (a C23 *ansa* chain includes actamycin, diastrovaricins, naphthomycins, naphthoquinomycins, naphthostatin, and naphthomycinol). The naphthomycin-type compounds are produced in most *Streptomyces* species with different substituents, such as −Cl, −H, −OH, −NR, or −SR groups at position 30 (Kang et al., 2012; Yang et al., 2012). These ansamycins display significant antimicrobial and antineoplastic activity, nevertheless showed weak antibiotic activities and cytotoxicities (Yang et al., 2018). Other napthtalenic ansamycins with relevant biological activities are described in Table 1.

**Table 1.** Relevant Traits of Diverse Napthalenic Ansamycins

Compound	Relevant chemical properties	Proposed mechanisms of action	Biological source	References
Streptovaricins	Contains a naphthoquinone nucleus and a macrolide aliphatic *ansa* bridge. Show identical structural skeleton with protorifamycins with a methyl group at the C-3 position of the naphthoquinone chromophore.	Inhibiting nucleoside incorporation and anti-leukemia virus by inhibiting ROSCHER leukemia virus RNA-dependent polymerase. Antibacterial activity against Gram-positive and Gram-negative bacteria, especially against *Mycobacterium tuberculosis* and methicillin-resistant *Staphylococcus aureus*.	*Streptomyces* sp. S012 *Streptomyces* sp. CS *Senna spectabilis* CCTCC M2017417	(Liu et al., 2017; Luo et al., 2021; Zhang et al., 2017)
Ansalactam A	Possess a γ-lactam residue with an aliphatic side chain, in which AHBA-derived amino group is spiro attached to the naphthalenic backbone. The C22–C27 aliphatic side chain of the lactam represents a novel polyketide biosynthetic building block.	Antibiotics currently used to the treatment of tuberculosis and leprosy, anticancer drug inhibiting heat-shock protein 90.	*Streptomyces* strain CNH189	(Hager et al., 2014; Kang et al., 2012; Le et al., 2016; Wilson et al., 2011)
Divergolides	Naphthoquinone ansamycin assembled via ring contraction of a macrocyclic precursor (proto-divergolide, both a macrolactone and a macrolactam). Divergolides possess additional linkages and highly strained *ansa* chains.	Displayed activity against *Bacillus subtilis* and *Mycobacterium vaccae.*	*Streptomyces* sp. HKIO576 and SCSGAA 0027 strains	(Nong et al., 2020; Terwilliger & Trauner, 2018; Wang et al., 2020)
Ansamitocins	Is a maytansinoid with 19-membered polyketide macrolide lactam.	Ansamitocin AP-3 is the most potent antitumor agent used as payload in many antibody conjugates, such as trastuzumab emtansine, FDA approved for breast cancer treatment. AP-3 can strongly depolymerize microtubule assembles in the mitotic cell phase cycle.	*Actinosynnema pretiosum* ATCC31565	(Du et al., 2017; Li et al., 2018; Liu et al., 2020)
Ansatrienin	Small molecules contain a 21-membered macrocyclic lactam ring and a cyclohexanoyl moiety attached via alanyl side chain attached to the C-11 hydroxyl group of the *ansa* ring.	Exhibit potent activity against fungi, yeasts, and cytotoxicity. Limited antibacterial activity.	*Streptomyces collinus, Streptomyces* sp. XZQH13	(Shi et al., 2016; Wang et al., 2018)

Mitomycin C is the most important member of mitomycins, with a relevant anticancer activity. Mitomycin C is used to treat different cancers such as head and neck sarcoma, lung carcinoma, bladder cancer, hepatic carcinoma, esophageal carcinoma, pancreatic, and colorectal or anal cancer (Baindara & Mandal, 2020; Bass et al., 2013; Wolters et al., 2021; Zhang et al., 2020). Mitomycin C is a potent DNA cross-linking alkylating agent by two postulated alkylating centers. The alkylation of DNA targets multiple guanine residues by reductive activation resulting in six covalent DNA adducts. DNA alkylation will block DNA synthesis, inhibiting cell proliferation, and several of the formed adducts are reported to induce cancer cell death (Wolters et al., 2021; Zhang et al., 2020). Mitomycin C possesses other relevant biological activities such as an antibiotic, antifibrotic, and immunosuppressive agent. However, severe adverse effects resulting from its high toxicity have limited its clinical application significantly (Zhang et al., 2020).

### ASA as the Precursor for Oseltamivir Phosphate Synthesis

Oseltamivir phosphate (Tamiflu) is one of the most potent oral anti-influenza neuraminidase inhibitors used to treat both seasonal and pandemic influenza (Sagandira et al., 2020; Tompa et al., 2021). Shikimic acid was initially used as the substrate for the chemical synthesis of Tamiflu by Gilead Sciences in 1995, codeveloped and marketed with F. Hoffmann-La Roche Ltd., and released commercially in 1999 (Sagandira et al., 2020). Although there are more than 70 OSP synthesis processes nowadays, Roche's industrial synthesis process from shikimate is the route that currently provides all OSP worldwide (Magano, 2009; Sagandira et al., 2020). Roche's industrial synthesis for OSP from SA utilizes the azide chemistry to incorporate a 1,2,-diamine moiety, but this process possesses critical steps, including the safety handle of the thermally unstable azide reagents and intermediates at a large scale (Sagandira et al., 2020)

As Roche's chemosynthetic route for OSP synthesis from SA involves the azide chemistry, the presence of an amino group at C5 in the ASA's molecule represents an advantage over SA as the substrate for the chemical synthesis of OSP, avoiding the requirement of azide chemistry to incorporate the 1,2,-diamine moiety in the aromatic ring, improving the synthesis of OSP and other oseltamivir carboxylates significantly (Diaz Quiroz et al., 2014; Frost & Guo, 2011; Magano, 2009).

Its extraction has ensured the SA supply of the synthesis of OSP from extensive plantations of Chinese star anise (mainly *Illicium religiosum*) and its production through fermentative processes using mainly engineered overproducing strains of *Escherichia coli* (Chandran et al., 2003; Diaz Quiroz et al., 2014; Martínez et al., 2015; Rodriguez et al., 2013; Sagandira et al., 2020). However, significant efforts are needed to produce ASA by biotechnological processes as a possible substrate for the chemical synthesis of OSF.

## Metabolic Engineering Strategies for the Microbial Production of ASA Pathway Intermediates Kanosamine, ASA, and AHBA

Given the relevance of kanosamine, ASA, and AHBA, intuitive metabolic engineering strategies to obtain native or heterologous overproducing bacterial strains have been proposed to overproduce these metabolites. Strategies for overproducing kanosamine should consider an increased availability of kanosamine or kanosamine-6-P by channeling glucose flux to their synthesis without affecting the glucose flux to the central carbon metabolism pathways (glycolysis and the PPP). As kanosamine shows antibiotic activity (Janiak & Milewski, 2018), it is necessary to avoid its intracellular accumulation through an efficient conversion to iminoE4 (Frost & Guo, 2011). Remarkably, the metabolic engineering strategies for the overproduction of ASA and AHBA also should avoid the competence of the SA pathway reaction for PEP (PEP + E4P → DAHP), and favoring its flow toward the synthesis of aminoDAHP through the ASA pathway (PEP + imoniE4P → aminoDAHP); otherwise, producing strains, particularly for overproduction of ASA purposes, should avoid SA contamination as resulted previously (Guo & Frost, 2004).

### Strategies for the Overproduction of Kanosamine

The kanosamine (3-amino-3-deoxy-D-glucopyranose) was first isolated as a byproduct of the acid hydrolysis of kanamycin but was further isolated from cultures of former *Bacillus aminoglycosides* (now *Bacillus pumillus*) (Umezawa, 1968; Umezawa et al., 1967). In *A. mediterranei*, the kanosamine is synthesized from UDP-glucose by six enzymatic reactions (Fig. 2A). The synthesis of kanosamine from glucose-6-P by three enzymatic reactions, was present and characterized in *Bacillus subtilis* UW85 and is encoded by the genes *kabABC*, encoding for similar enzymes encoded by the *ntdABC* operon in *B. subtilis* (Prasertanan & Palmer, 2019; Vetter et al., 2013) (Fig. 2B). *B. pumillus* ATCC 21143 was reported as a higher natural producer of kanosamine from D-glucose, resulting in titers up to 20 g/l in 28% mol/mol yield from glucose (Table 2) (Guo & Frost, 2004).

**Table 2.** Natural and Engineered Derivative Bacterial Strains for the Overproduction of Kanosamine, ASA, and ADHA

Naturally producing or derivative strains	Target ASA pathway intermediate	Phenotypic traits	Culture conditions	Titer and yield (mol/mol from glucose)	References
*Bacillus pumillus* ATCC 21143	Kanosamine	Wild-type strain	Glucose as the carbon source, soybean or peanut meal as the nitrogen source, in fermentor-controlled conditions	25 g/l, 28%	(Guo & Frost, 2004)
*Escherichia coli* RB791*serA*	Kanosamine	pSN1.139/p*trc ndcA ntdB ntdC serA*	Glucose-rich and glucose-limited fed-batch fermentation	12.7–18 g/l, 6%	(Miller, 2018)
*Streptomyces coelicolor* YU105/pHGF7612	AHBA	pHGF7612/actII-orf4 *rifJNMLKGHI*	R5 medium (without sucrose)	0.35–0.5 g/l	(Yu et al., 2001)
*E. coli* BL21/pKW256	AHBA	pKW256/p*T7rifH rifM asm23 asm24, fifL firm rifK pccB accAI*	M9 medium containing kanamycin 50 μg/ml, 1 mM IPTG, shake flask cultures	3.1 mg/l	(Watanabe et al., 2003)
*E. coli* BAPI/pKW255	AHBA	pKW255	LB supplemented with IPTG	10 mg/ml	(Rude & Khosla, 2006)
*Amycolatopsis mediterranei* ATCC 21789/pJG8.219A	ASA	pJG8.219A/p*amy rifI amy ermE* Kam^R^/Neo^R^	1.5% soybean meal as the nitrogen source, 1.0% glucose as the carbon source, 0.3 Nacl, 20°C, Shake flask cultures	0.2 g/l	(Guo & Frost, 2004)
*E. coli* SPI.1/pJG5.166A	ASA	pJGJ5.166A/p*tac rifH*^2^ p*tac aroE*^3^*tktA*^3^*serA^3^* Amp^R^	1.5% soybean meal, 1.0% glucose as the carbon source, 0.3 Nacl, 20ºC, Flask shake cultures	0.81 g/l ASA + 3.7 g/l SA	(Guo & Frost, 2004)
Coculture of *Bacillus pumillus* ATCC 21143 with *E. coli* SPI.1/pJG5.166A	Kanosamine (*B. pumillus*) ASA (*E. coli*)	pJGJ5.166A/p*tac rifH*^2^ p*tac aroE*^3^*tktA*^3^*serA^3^* Amp^R^	Glucose as the carbon source, soybean or peanut meal as the nitrogen source, and fermentor-controlled conditions	Up to 25 g/l of kanosamine (*B. pumillus*); 1.1 g/l ASA + 3.4 g/l SA (*E. coli*)	(Guo & Frost, 2004)

^a^ *rifH* locus from *Amycolatopsis mediterranei; ^b^aroE* and *tktA* genes from *Escherichia coli*.

The heterologous expression of the kanosamine biosynthetic pathway from *A. mediterranei, B. subtilis*, and *B. pumillus* in *E. coli* was explored to determine if the heterologous system could be manipulated to maximize kanosamine production. Recombinant *E. coli* PSNI.39 carrying the plasmid pSN1.292/*ntdCAB* with the *ntd*ABC genes from *B. subtilis* 168 produced 12.7 g/l kanosamine in 6% mol/mol yield from glucose, showing a further increment to 18 g/l by blocking the glycolytic pathway by a mutation in the *E. coli* housekeeping *pgi*-encoded phosphoglucose isomerase. Remarkably, the production of kanosamine resulted in the accumulation of L-glutamic acid in supernatant cultures of the *pgi*^–^ mutant. This result suggested that increased L-glutamate accumulation could have a beneficial effect on the heterologous production of kanosamine in *E. coli* expressing the *Bacillus ntd* biosynthetic genes, as L-glutamate is the cosubstrate for NtdA (pyridoxal phosphate-dependent 3-oxo-glucose-6-phosphate: glutamate aminotransferase) (Fig. 2B) (Miller, 2018).

### Strategies for the Overproduction of AHBA for the Biosynthesis of Ansamycins

The biosynthesis of the naphtalenic ansamycins is a complex biosynthetic process starting with the synthesis of the precursor AHBA in the ASA pathway, and it is further loaded onto a type I PKS, where several rounds of elongation occur, and the final polyketide cycling and modification of the chain yield a macrocyclic lactam (Kang et al., 2012; Wang et al., 2017). The importance of the intracellular availability of AHBA in the production of ansamycin polyketides such as rifamycin and geldanamycin was demonstrated in engineered derivatives of *Streptomyces hygroscopicus* XM201. The transcriptomic analysis of the producing mutant led to identifying that PKS genes *gdmA1-A3* were downregulated compared to that involved in AHBA biosynthesis and postmodifications. Upregulation of PKS genes under a strong promoter (5063p) increased its expression significantly and resulted in a 39% increment in geldanamycin yield. Nevertheless, exogenous feed of AHBA showed that this precursor was now the rate-limiting step in the biosynthetic process. Overexpression of the AHBA biosynthetic cluster (*orf990-orf995*) assembled under the strong promoter 5063p increased geldanamycin yield both in the wild-type and derivative strains. The combined expression of PKS and AHBA biosynthetic cassettes increased the yield of geldanamycin by 88% in the producing strains compared to the wild-type strain, highlighting the relevance in the availability of AHBA in the engineered strains (Wang et al., 2017).

The elucidation of the transcriptional control mechanisms of the *rif* cluster for the synthesis of rifamycin in *A. mediterranei* by GlnR, the global nitrogen regulator in this bacterium, showed that GlnR binds specifically to the upstream region of *rifZ*, encoding for the RifZ specific activator of the *rif* cluster, acting as an indirect regulator of the entire biosynthetic cluster. Furthermore, GlnR was also determined to bind to the upstream region of *rifK*, coding for the AHBA synthase, acting as a direct activator of the supply of AHBA for the synthesis of rifamycin (Liu et al., 2020c) (Fig. 4B).

The heterologous expression of AHBA biosynthetic genes in *E. coli* resulted in the successful production of this precursor unit for the potential synthesis of diverse ansamycins and remarkably as a source of amine-substituted SA derivatives. A heterologous hybrid AHBA biosynthetic pathway expressed in *E. coli* BAP1 includes *rifF, rifH, rifK, rifL, rifM*, and *rifN* genes; the bicistronic RifA construct and the *pccB* and a*ccA1* genes from *S. coelicolor.* Cultures of this derivative strain produced 2,6-dimethyl-3,5,7-trihydroxy-7-(3´-amino-5´-hydroxyphenyl)-2,4-heptadienoic acid (P8/1–09), an intermediate of the rifamycin biosynthetic pathway, AHBA; and the amine-derivatives aminoDAHP and aminoDHS. These results provided a relevant basis for further heterologous production and manipulation of AHBA-derived polyketides and intermediates of the aminoSA pathway in *E. coli* (Watanabe et al., 2003).

### Strategies for the Overproduction of ASA

Guo and Frost (2004) reported the first microbial production of ASA from glucose as the carbon source in cultures of *A. mediterranei* ATCC 21789 as in *E. coli* SP1.1. Cloning of the *rifI* gene from *A. mediterranei* in the plasmid PJG8.219A/p*amy rifI amy ermE kan neo* (Table 2) and transformation into this bacterium resulted in the production of 0.2 g/l of ASA in 0.4 mol/mol yield from glucose and 1.4 g/l of rifamycin B under controlled culture conditions. Further derivatives of *E. coli* SP1.1 *aroKL*^–^ (previously used as the genetic host for the production of SA [Chandran et al., 2003]) were transformed with plasmid pJG5.166A/*rifI aroE tktA* (Table 2) and cultures of this derivative strain under controlled fermentation conditions with the external addition of kanosamine resulted in the production of 0.81 g/l ASA and 3.7 g/l SA. Finally, these authors explored a coculture with *B. pumillus* ATCC 21143 and *E. coli* SP1.1 *aroKL*^–^/pJG6.181B *rifI aroE tktA glk* (Table 2). In this system, *B. pumillus* produced kanosamine from glucose-6-P to the culture medium and internalized by *E. coli* SP1.1 to produce ASA by the sequential intracellular phosphorylation of kanosamine isomerization kanosamine 6-phosphate and fragmentation of aminoF6P to form iminoE4P. Further condensation of PEP with iminoE4P by the native DAHP synthase isoenzymes of *E. coli* (*aroF, aroG, aroH*) results in aminoDAHP, which was sequentially converted to ASA by the enzymes of the SA pathway of *E. coli* AroB (3-dehydroquinate synthase, *aroB*), AroD (DHAQ dehydratase, *aroD*), and AroE (shikimate dehydrogenase, aro*E*) resulting in the production of 1.1 g/l of ASA and 3.4 g/l of SA (Guo & Frost, 2004). Given the relevance of ASA as a possible starting material in the synthesis of OSF antiviral for the treatment of seasonal and pandemic influenza, the optimization of the synthesis process in *E. coli* or some other host (e.g., *Corynebacterium glutamicum* [Kogure et al., 2016]) is of great relevance.

### The Impact of the Heterologous Expression of Non-ASA Pathway Genes in the Synthesis of Rifamycins

When the liquid cultures of *A. mediterranei* produce rifamycins reach the stationary phase, it exhibits a high viscosity associated with filamentous bacteria growth. This behavior results in the accumulation of unwanted rifamycins such as rifamycin W in supernatant cultures. The heterologous expression of the hemoglobin encoding gene *vhb* from *Vitreoscilla stercoraria* under the control of the PermE promoter in the *A. lactamdurans* plasmid pULVK2 in *A. mediterranei* resulted in the enrichment in the production of rifamycin B instead of the unwanted rifamycin W under low aeration conditions by increasing the oxygen-dependent production of rifamycin B. Addition of barbital to cultures of *A. mediterranei* promotes the production of rifamycin B exclusively. The expression of the hemoglobin *vhb* gene under low aeration cultures of *A. mediterranei* resulted in a 13.9% higher production of rifamycin B in cultures with barbital compared to the parental strain and increased to 29.5% without barbital (Priscila et al., 2008).

Further inclusion of the *vhb* gene bounded to cytochrome P450 (*rif-orf5*) gene from rifamycin biosynthetic cluster of *A. mediterranei* increased 1.5-fold higher rifamycin B production than the transformant with only the *vhb* gene and 2.2-fold higher than the parental strain. The expression of fused genes *vhb*-*orf5* facilitated oxygen availability for the limiting steps for the synthesis of rifamycin B (Mejía et al., 2018).

## Conclusion

As many metabolic aromatic intermediates and final compounds produced by biosynthetic processes show broad biological activity of pharmaceutical relevance such as antibiotics, anticancer agents, and enzyme inhibitors, it is relevant to understand the biosynthetic and control mechanisms involved in its production, particularly in microbial strains with prospects for industrial production. The ASA pathway has been extensively studied because AHBA is the key precursor for synthesizing a great diversity of secondary metabolites such as ansamycins and mitomycins. The relevance of the availability of AHBA as a key structural unit for the synthesis of ansamycins and mitomycins has been demonstrated by applying different omics such as genomics, transcriptomics, metabolomics, and fluxomics (Kogure et al., 2016; Liu et al., 2020b; Wang et al., 2017; Zhang et al., 2017). Several metabolic engineering strategies have been developed to increase the availability of AHBA in several species of *Streptomyces* and *A. mediterranei*, resulting in the efficient optimization in the production of target compounds such as ansamycins, geldanamycin, and rifamycins. Remarkably, the successful heterologous expression of AHBA biosynthetic genes in *E. coli* has provided the basis for further genetic and metabolic manipulations of AHBA-derived polyketides. Additionally, the construction of genome-scale models resulting from omic studies, its validation by predicting microbial growth under controlled conditions, and evaluating the role of critical genes in the synthesis of compounds such as ansamitocins (T. Liu et al., 2020b) could provide a valuable basis for the rational modification of the biosynthetic metabolism of AHBA and further elongation and modification steps in the biosynthesis of ansamycins and mitomycins antibiotics.

Another highly relevant intermediate of the ASA pathway is aminoshikimate. The relevance of this compound, particularly as a chemical precursor for the synthesis of OSP, the potent neuraminidase inhibitor of seasonal and pandemic influenza viruses, over SA, and its use in the synthesis of other oseltamivir carboxylates. Its production is particularly relevant because influenza viruses are the third most studied viruses after SARS and HIV ones, for the relevant implications in public health (Tompa et al., 2021). The application of metabolic engineering strategies for its production should consider the efficient production of the key precursor kanosamine and its efficient conversion to aminoDAHP instead of DAHP to avoid SA contamination. Possible strategies of cocultures with kanosamine producing *Bacillus* strains and selecting proper engineered *E. coli* hosts to produce ASA (avoiding the production of SA) could improve the valuable aromatic compound as the substrate for the chemical synthesis of OSP.

## Notes

**This contribution is dedicated to the memory of Professor Arnold L. Demain, for his valuable contributions in the field of industrial microbiology, on the production of secondary metabolites, and for his committed work in the training of many generations of human resources in the academy and the industry.**
